# Treatment for chemotherapy-induced peripheral neuropathy: A systematic review of randomized control trials

**DOI:** 10.3389/fphar.2022.1080888

**Published:** 2022-12-23

**Authors:** Chenkun Wang, Si Chen, Weiwei Jiang

**Affiliations:** ^1^ Department of Pharmacy, The Second Affiliated Hospital, Chongqing Medical University, Chongqing, China; ^2^ College of Pharmacy, Chongqing Medical University, Chongqing, China; ^3^ Department of Orthopedics, The Second Affiliated Hospital, Chongqing Medical University, Chongqing, China

**Keywords:** chemotherapy-induced peripheral neuropathy (CIPN), rrandomised controlled trial, drugs, treatment, efficacy, safety, systematic review

## Abstract

**Purpose:** Treatment of chemotherapy-induced peripheral neuropathy (CIPN) is challenging for clinicians, and many clinical trials and meta-analyses on CIPN are controversial. There are also few comparisons of the efficacy among drugs used to treat CIPN. Therefore, this systematic review aimed to study the efficacy of drugs in treating CIPN using existing randomized controlled trials.

**Methods:** Electronic databases were searched for randomized controlled trials (RCTs) involving any pharmaceutical intervention and/or combination therapy of treating CIPN.

**Results:** Seventeen RCTs investigating 16 drug categories, duloxetine, pregabalin, crocin, tetrodotoxin, venlafaxine, monosialotetrahexosyl ganglioside (GM1), lamotrigine, KA (ketamine and amitriptyline) cream, nortriptyline, amitriptyline, topical *Citrullus colocynthis* (bitter apple) oil, BAK (baclofen, amitriptyline hydrochloride, and ketamine) pluronic lecithin organogel, gabapentin, and acetyl l-carnitine (ALC), in the treatment of CIPN were retrieved. Many of the included RCTs consisted of small sample sizes and short follow-up periods. It was difficult to quantify due to the highly variable nature of outcome indicators.

**Conclusion:** Duloxetine, venlafaxine, pregabalin, crocin, tetrodotoxin, and monosialotetrahexosyl ganglioside exhibited some beneficial effects in treating CIPN. Duloxetine, GM1, and crocin showed moderate benefits based on the evidence review, while lamotrigine, KA cream, nortriptyline, amitriptyline, and topical *Citrullus colocynthis* (bitter apple) oil were not beneficial. Further studies were necessary to confirm the efficacy of gabapentin in the treatment of CIPN because of the controversy of efficacy of gabapentin. Furthermore, BAK topicalcompound analgesic gel only had a tendency to improve the CIPN symptoms, but the difference was not statistically significant. ALC might result in worsening CIPN. Most studies were not of good quality because of small sample sizes. Therefore, standardized randomized controlled trials with large samples were needed to critically assess the effectiveness of these drugs in treating CIPN in the future.

## 1 Introduction

Chemotherapy-induced peripheral neuropathy (CIPN) is one of the main dose-limiting side effects of neurotoxic anticancer drugs. The chemotherapy dose needs to be reduced or completely paused when CIPN develops. All of the commonly used chemotherapeutic drugs such as taxanes, platinum derivatives, vinca alkaloids, thalidomide, and bortezomib all can cause CIPN (Staff et al., 2017; Shah et al., 2018; Colvin, 2019). Regarding overall neurotoxic chemotherapy, after completion of chemotherapy, the incidence of CIPN was approximately 68% after 1 month, 60% after 3 months, and 30% after 6 months and above (Colvin, 2019). For a particular chemotherapy drug, the incidence of CIPN varied among many previous reports. However, taxanes and platinum derivatives were the most prone to develop CIPN (Shah et al., 2018; Colvin, 2019). CIPN is mainly characterized by sensory nerve symptoms, presenting with glove and stocking pain, and patients often report numbness, tingling, and pain. CIPN can also be accompanied by motor or autonomic nerve symptoms (Loprinzi et al., 2020). Additional medications or other interventional measures are often required to treat these symptoms that otherwise seriously affect the patient’s quality of life, and these remedial measures cause financial burdens on the patients (Miaskowski et al., 2018). The average monthly drug treatment costs for CIPN ranged from USD 15 to USD 1425. Among duloxetine, gabapentin, pregabalin, amitriptyline, nortriptyline, and venlafaxine, the average monthly costs of duloxetine ranged from USD 241 to USD 637 (Gupta et al., 2022). It is worth mentioning that duloxetine is the only drug recommended for painful CIPN (intermediate evidence quality, moderate strength of recommendation), and no agents are recommended for the prevention of CIPN, suggested by American society of clinical oncology (ASCO) guidelines (Loprinzi et al., 2020). The treatment of CIPN is a significant issue, but numerous existing clinical trials and meta-analyses on the treatment of CIPN are still controversial. Furthermore, head-to-head clinical trials are rare. It is urgent to evaluate and find out new and superior drugs in treating CIPN since the evidence is scanty in comparing their efficacy. Therefore, this systematic review aimed to estimate the efficacy of drugs in treating CIPN to provide a reference for clinical practice and future research.

## 2 Material and methods

This systematic review was performed following the Preferred Reporting Items for Systematic Review and Meta-Analysis Protocols (Moher et al., 2015). The protocol is available in the PROSPERO database (ID number: CRD42022334388). Although we initially planned to conduct systematic review and meta-analysis, we only conducted systematic review due to the variability in outcome indicators and small sample size in each drug category.

### 2.1 Search strategies and selection criteria

We searched the PubMed, EMBASE, and Cochrane library databases from their inception to 20 March 2022. Then, additional electronic database searches were conducted to obtain comprehensive and up-to-date information, up to 31 August 2022. We used the Medical Subject Headings (Mesh) and their free words for “chemotherapy”, “peripheral neuropathy”, and “randomized controlled trial”, and their respective subject terms and free words were linked by “OR”, followed by “And”, and the title and abstract were searched.

Open or blinded studies have been included and all included trials met the following criteria: (1) Adult patients (age of ≥18 years); (2) Patients who developed chemotherapy-induced peripheral neuropathy; (3) CIPN with any single drug intervention and/or combination of drugs administration; and (4) randomized controlled trials (RCTs). The following criteria were used for exclusion: (1) Patients with other neuropathic diseases such as diabetes, acquired immune deficiency syndrome (AIDS/HIV), vitamin B12 deficiency, and serious mental disease; (2) Patients who underwent traditional Chinese medicine decoction and physical therapy; (3) Studies with non-human subjects and non-RCT design; (5) Duplicate studies, reviews, systematic reviews and meta-analyses, and case reports and case series; (6) Publications in non-English. Two authors independently screened and then comprehensively reviewed the titles, abstracts, and articles. Any disagreement between reviewers was resolved by consensus in all cases. The authors of the incomplete studies were contacted by email, but we did not receive a response.

### 2.2 Data extraction

Two researchers independently extracted relevant data from the RCTs (Table 1). If there was any disagreement with the data, they negotiated to reach a consensus. If the literature was unavailable or the data was lacking, we would try our best to contact the author to obtain related resources. If the outcome indicators were only shown in a graphical presentation, Engauge Digitizer software was used to extract the data.

**TABLE 1:** Extracted data characteristics.

Basic information of Included Trials	First Author and year of Publication
Characteristics of the research subjects	Total number of participants and the number of each group, gender of patients in each group, and age (mean ± SD)
Intervention	Chemotherapeutic dosage, course of treatment, and others
Key elements of bias risk	RandomACT sequence generation, blinding, allocation concealment, completeness of outcome data, selective reporting, measurement bias, and other bias
Outcome	All outcome indicators in each study such as average change of pain score (mean ± SD), quality of life score, and others

### 2.3 Risk assessment of bias

Two researchers used the Cochrane collaboration’s risk of bias tool to determine the bias risk of all included randomized controlled trials (Higgins et al., 2011). The following seven items were evaluated, including random sequence generation, blinding of participants and personnel, blinding of outcome assessment, allocation concealment, incomplete outcome data, selective reporting, and other bias. The result of the evaluation on each item was “low risk”, “unclear risk”, or “high risk”. In case of a disagreement, the two executors reached a consensus through negotiation and discussion. If the dispute was still unresolved, the decision was made by a third party.

### 2.4 Assessment of evidence quality

The Grading of Recommendations Assessment, Development, and Evaluation (GRADE) approach was used to evaluate the quality or certainty of each compared evidence (Salanti et al., 2014). The five degraded factors in the GRADE assessment were risk of bias, inconsistency, indirectness, imprecision, and publication bias. The three upgraded factors in the GRADE assessment were large effect, plausible confounding would change the effect, and dose response gradi. The recommended quality of the final report was divided into high, medium, low, and extremely low. Moreover, the recommended strength was divided into strong and weak. The evidence of high quality indicates that the effect value is very close to the actual value, a strong recommendation indicates that the advantages outweigh the disadvantages, and a weak recommendation means that the disadvantages outweigh the advantages.

### 2.5 Outcome

The changes in pain and neuropathic symptoms were our primary measures of concern, but all outcome measures are summarized for each study due to variations in outcome measures among RCTs.

## 3 Results

### 3.1 Study selection

**FIGURE 1:**
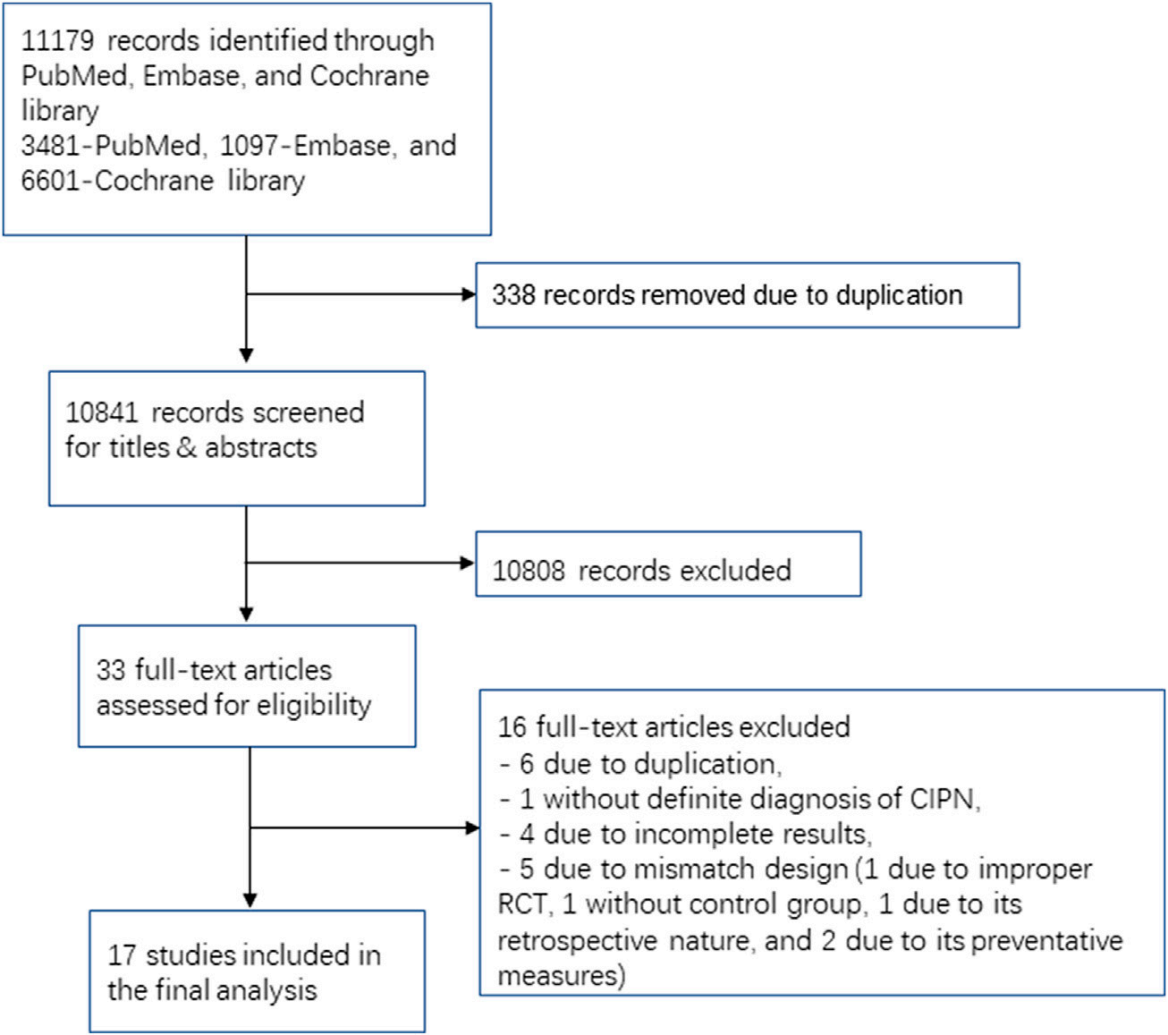
Flow diagram.

Seventeen RCTs involving 16 medication classes ultimately met the inclusion criteria (Table 2 and Figure 1). Among these RCTs, five studies were conducted with duloxetine, three trials were carried out with pregabalin, and two studies were conducted with gabapentin. Lamotrigine, crocin, tetrodotoxin, nortriptyline, amitriptyline, acetyl l-carnitine, venlafaxine, GM1 (Monosialotetrahexosyl ganglioside), KA (ketamine and amitriptyline) cream, BAK (baclofen, amitriptyline, and ketamine) pluronic lecithin organogel, and topical *C. colocynthis* oil were investigated in one study each.

**TABLE 2:** The type of therapeutics and the number of relevant RCTs.

	Type of treatments	The number of relevant RCTs
C	Crocin	1
D	Duloxetine	5
G	Gabapentin	2
L	Lamotrigine	1
KA	Ketamine plus Amitriptyline	1
P	Placebo	13
Pg	Pregabalin	3
T	Tetrodotoxin	1
VB_12_	Vitamin B_12_	1
N	Nortriptyline	1
A	Amitriptyline	1
BAK	Baclofen plus Amitriptyline plus Ketamine	1
ALC	Acetyl l-Carnitine	1
V	Venlafaxine	1
TC	Topical *C. colocynthis* oil	1
GM1	Monosialotetrahexosyl ganglioside	1

### 3.2 Study characteristics

**FIGURE 2:**
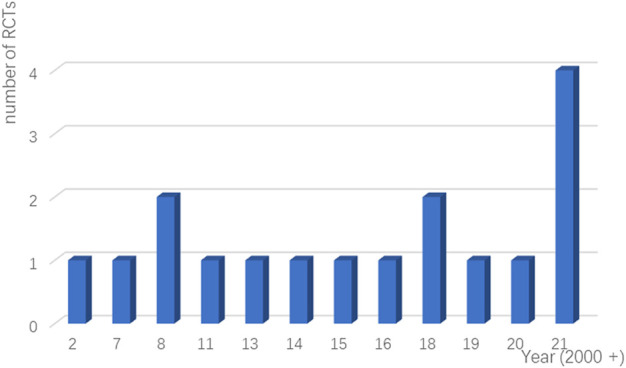
Number of RCTs on CIPN treatment published each year from 2007 to 2021.

Seventeen RCTs from seven countries were included in the final analysis and the average age was 60 years. The studies were published from 2007 to 2021 (Figure 2), and the lowest score of the impact factor was 1.4 and the highest score was 51 [the first RCT to demonstrate the therapeutic effect of duloxetine for CIPN published in 2013 (Smith et al., 2013)]. The sample sizes ranged from 32 to 462. Additionally, intervention drugs were gabapentin, lamotrigine, duloxetine, venlafaxine, ketamine and amitriptyline (KA) cream, vitamin B12, crocin tablets, tetrodotoxin, nortriptyline, baclofen, amitriptyline hydrochloride and ketamine pluronic lecithin organogel (BAK-PLO), acetyl l-Carnitine (ALC), pregabalin, topical *C. colocynthis* oil, and monosialotetrahexosyl ganglioside (GM1). Most patients developed peripheral neuropathy caused by platinum or taxane. The basic characteristics of patients and reasons for inclusion are detailed in Table 3.

**TABLE 3:** Basic characteristics of the subjects and information for inclusion.

Included Studies	Country	Mean/Median Age (years) (Range/SD)		Sex (male/Female)		Patients	Anticancer drugs
		Treatment	Control	Treatment	Control		
Rao et al. (2007)	United States	59 (28–84)	60 (25–80)	15/42	16/42	Patients, with CIPN, whose duration of ≥1 month	Taxanes, platinum compounds, and vinca alkaloids
Rao et al. (2008)	United States	62 (29–84)	59 (34–82)	27/36	24/38	Patients, with CIPN, whose duration of ≥1 month	Taxanes, platinum compounds, and vinca alkaloids
Smith et al. (2013)	Philippines	60 (10.4)	59 (10.6)	38/71	44/67	CIPN patients (sensory neuropathy of ≥grade 1 and pain score of ≥4)	Taxanes, platinum
Gewandter et al. (2014)	United States	NA	NA	73/156	62/171	Patients with CIPN, pain score of ≥4 and Karnofsky performance status of >60	Taxanes 246 (53%)
Hirayama et al. (2015)	JPN	61 (48–7)	64 (49–75)	8/9	9/8	Patients with CIPN, sensory neuropathy of >1 and pain score of ≥4	Taxanes and platinum
Manjushree et al., 2021	India	50.6 (12)	53 (7.6)	9/24	9/21	Patients with CIPN	Paclitaxel, carboplatin, bortezomib, thalidomide, vincristine, oxaliplatin, cisplatin
Bozorgi et al. (2021)	Iran	61 (27–84)	62 (25–89)	41/48	42/46	Patients, with CIPN, pain scores≥4 and duration≥1 month	Taxanes, platinum compounds, and vinca alkaloids
Goldlust et al. (2021)	United States	60.6 (11.1)	59 (1.5)	10/16	10/15	patients with CIPN	Taxanes and platinum compounds
Hammack et al. (2002)	United States	58.7	58.7	NA	NA	CIPN patients with paresthesia and pain for at least 1 month	Cis-platinum
Kautio et al. (2008)	Finland	52 (37–67)	54 (35–67)	3/14	5/11	Patients with neuropathy presenting with numbness, tingling, or with pain score of ≥3	Taxus, platinum, or vinblastine
Barton et al. (2011)	United States	59.9 (10.75)	62.1 (10.27)	35/66	42/60	Patients with CIPN, symptom duration of >1 month	Taxus, platinum, vinblastine, thalidomide and other drugs
SUN et al. (2016)	China	NA	NA	NA	NA	Patients with CIPN, symptom duration of ≥1 month	Paclitaxel, cisplatin, or vinblastine
Avan et al. (2018)	Iran	Pregabalin, NA (29–72)	Duloxetine, NA (30–71)	NA	NA	Breast cancer patients with sensory neuropathy and pain score of ≥4	Taxane
Farshchian et al. (2018)	Iran	Duloxetine, 63.85 (7.58)	Duloxetine 15/37	Duloxetine 15/37	Placebo 10/42	Patients with CIPN	Taxane and Platinum
		Venlafaxine, 57.44 (14.53)	Venlafaxine 7/45	Venlafaxine 7/45			
Rostami et al. (2019)	Iran	59.23 (13.08)	55.25 (11.19)	5/12	6/9	Cancer patients diagnosed by neurologist as peripheral neuropathy and received chemotherapy over the previous 2 months	Taxane/oxaliplatin
Salehifar et al. (2020)	Iran	49.4 (9.67)	48.7 (9.63)	Pregabalin, total 40	Duloxetine, total 42	Patients (sensory neuropathy of ≥grade 1 and VAS score of ≥4)	Paclitaxel or docetaxel
Zhou et al. (2021)	China	60 (23–79)	60 (24–75)	52/21	45/27	Patients with chronic OIPN	Oxaliplatin

### 3.3 Risk of bias and quality of evidence assessments

**FIGURE 3:**
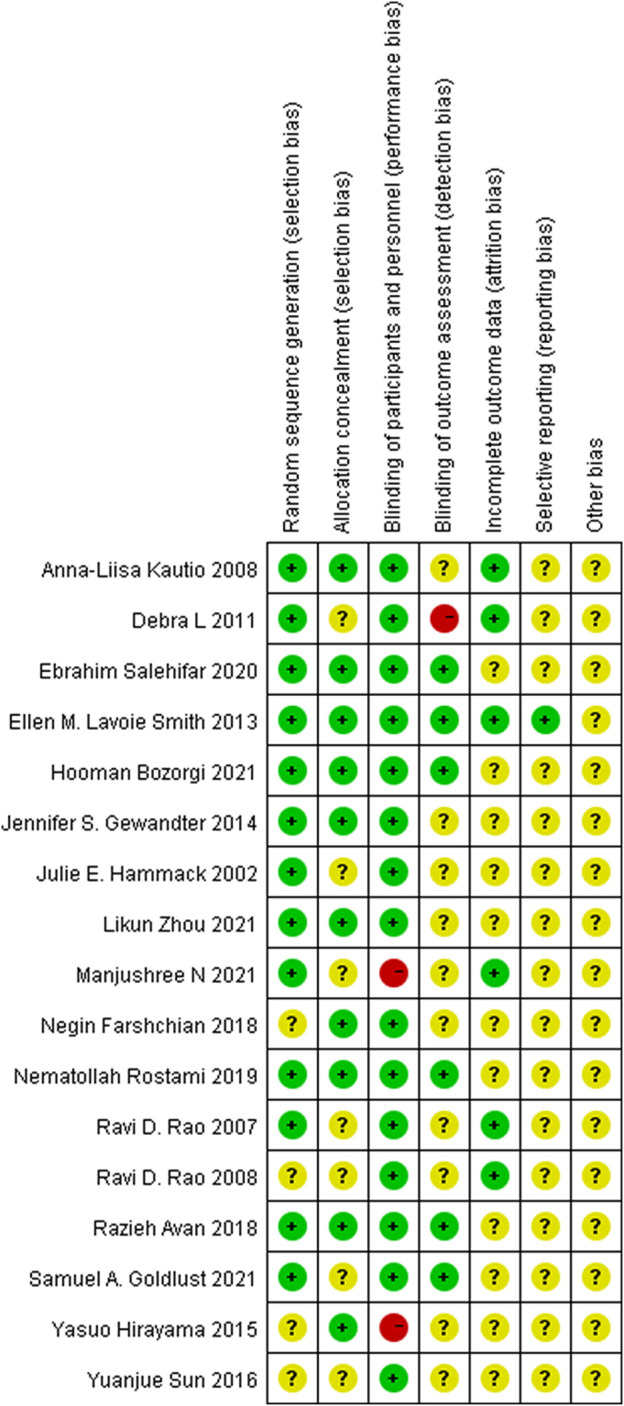
Risk of bias summary.

Most of RCTs included in the analysis had a low risk of bias. Two studies showed high-risk bias due to poor blinding of participants and other personnel involved in the trial (performance bias), and only one study had a high risk due to inadequate blinding of outcome assessment (detection bias). The details are shown in Figure 3.

Upon assessing the results for the quality of evidence using the GRADE approach, most of the evidence was of very low or low quality (Table 4).

**TABLE 4:** Grade Assessment.

Comparison	Downgrade quality of evidence					Upgrade quality of evidence		
	Risk of bias	Inconsistency	Indirectness	Imprecision	Publication bias	Large effect	Plausible confounding would change the effect	Dose response gradi
Very low								
G vs. P	S	N	N	VS	Un	N	N	N
L vs. P	S	N	N	VS	Un	N	N	N
KA vs. P	N	N	N	VS	Un	N	N	N
G vs. Pg	S	N	N	VS	Un	N	N	N
T vs. P	S	N	N	VS	Un	N	N	N
BAK vs. P	S	N	N	VS	Un	N	N	N
D vs. Pg	S	S	S	VS	Un	N	N	N
Low								
D vs. VB12	S	N	N	VS	Un	VL	N	N
N vs. P	N	N	N	VS	Un	N	N	N
A vs. P	N	N	N	VS	Un	N	N	N
TC vs. P	N	N	N	VS	Un	N	N	N
ALC vs. P	S	N	N	VS	Un	L	N	N
V vs. P	N	N	N	VS	Un	N	N	N
Moderate								
D vs. P	N	N	N	VS	Un	L	N	N
C vs. P	N	N	N	VS	Un	L	N	N
GM1 vs. P	N	N	N	VS	Un	L	N	N

N: No; S: Serious; VS: Very Serious; Un: Undetected; L: Large; VL: Very Large.

N: +0; S: 1; VS: 2; Un: +0; L: +1; VL: +2.

### 3.4 Study results

Five studies did not demonstrate any effectiveness for lamotrigine, KA cream, nortriptyline, amitriptyline, and topical *C. colocynthis* oil. A study on BAK (baclofen, amitriptyline, and ketamine) pluronic lecithin organogel found that the sensory neuropathy subscale was improved but without statistical significance. However, duloxetine, venlafaxine, pregabalin, crocin, tetrodotoxin, acetyl l-carnitine, and monosialotetrahexosyl ganglioside were effective for CIPN. A subsequent study on ALC (Hershman et al., 2018) found that long-term (24 weeks) ALC treatment worsened the CIPN over 2 years. Furthermore, the efficacy of gabapentin in the treatment of CIPN was disputed in two studies. The detailed study results such as intervention, duration of intervention, and outcome indicators are summarized in Supplementary Table S1.

#### 3.4.1 Gabapentin

A study conducted in the United States in 2007 (Rao et al., 2007) and a study conducted in India in 2021 (Kim et al., 2018) investigated the effect of gabapentin in treating CIPN. However, the results from these two studies were debatable. The former reported no benefit with gabapentin treatment, while the latter showed benefit with the same treatment. All indicators such as the symptoms of pain, the quality of life, and the WHO neuropathy score did not statistically differ from those of the placebo group in the former study, whereas gabapentin and pregabalin improved the pain caused by CIPN in the latter study. After 8 weeks of treatment of, the VAS decreased from 8.3 ± 1.43 to 1.8 ± 2.51 (*p* < 0.0001) in gabapentin and from 8.2 ± 1.62 to 0.8 ± 0.96 (*p* < 0.0001) in pregabalin. Pain quality assessment scale (PQAS) score reduced from 34.8 ± 6.67 at baseline to 10.2 ± 10.96 after gabapentin treatment and from 36.9 ± 8.5 to 4.5 ± 3.66 in the pregabalin arm (*p* < 0.0001). Furthermore, 2 (6.06%) patients in the gabapentin group and 1 (3.33%) patient in the pregabalin arm required rescue medications. A summary of the information is shown in Table 5. In Rao et al., 2007, no difference in the incidence of adverse events was found between the treatment and placebo groups. In Kim et al., 2018, the incidence of adverse events was higher with gabapentin (21.1%) treatment than with pregabalin (16.6%) treatment. Meanwhile, sedation (6.60%), drowsiness (9.09%), and diplopia and blurring of vision (3.03%) were the common adverse events in the gabapentin arm, whereas adverse events, sedation (13.3%) and drowsiness (3.3%) frequently occurred in the pregabalin group.

**TABLE 5:** The summary of two studies about gabapentin.

Basic information of included studies	Country	Rao et al. (2007)	Manjushree et al., 2021
		United States	India
Participants	Mean Age (years	Gabapentin: 59 (28–84)	Gabapentin: 50.6 ± 12
	(Range/SD)	Placebo: 60 (25–80)	Pregabalin: 53 ± 7.6
	The total sample size	115	63
	Sex (Male/Female)	Gabapentin: 15/42	Gabapentin: 9/24
		Placebo: 16/42	Pregabalin: 9/21
	Type of chemotherapy	Taxanes (40%), platinum compounds (21%), and vinca alkaloids	Taxanes (79.35%), platinum compounds, vinca alkaloids bortezomib, and thalidomide
		(Combined chemotherapy accounted for 13%)	
Intervention	Dosage of administration	Gabapentin capsules were started at 300 mg/d and increased to 2700 mg/d within 3 weeks	Gabapentin, 300 mg, bid, P.O.
			Pregabalin 75 mg, bid, P.O.
	Intervention time (Weeks)	14 (2W)	8
	(Washout period)		
Comparison		Placebo	Pregabalin
Outcome		Gabapentin was not beneficial in the treatment of CIPN (See Supplementary Table S1 for details)	Both gabapentin and pregabalin relieved pain symptoms of CIPN(See Supplementary Table S1 for details)
Study design		RCT, open-label, crossover study	RCT, open-label study

#### 3.4.2 Lamotrigine

Lamotrigine was investigated in a 10-week double-blinded RCT with a total sample size of 125, conducted in 2008 in the United States (Rao et al., 2008). CIPN symptoms with ≥ 1-month duration, caused by taxanes, platinum compounds, and vinca alkaloids were treated with lamotrigine, and the dose was gradually increased from 25 mg to 150 mg. However, pain, depression, and quality of life were not improved, and lamotrigine treatment was ineffective for patients with CIPN. There was no statistically significant difference in adverse events between the two arms. The most common adverse events were ataxia, rash, constipation, arthralgia, gastrointestinal reaction, pruritis, fatigue, and headache.

#### 3.4.3 Nortriptyline

Nortriptyline was investigated in a double-blinded, randomized, controlled, crossover study in the treatment of platinum-induced peripheral neuropathy, and this study was conducted in the United States in 2002 (Hammack et al., 2002). The sample size was 91, and the treatment lasted for 9 weeks. Nortriptyline tablets started at a dose of 25 mg/d and increased by 25 mg/d every other week to a maximum dose of 100 mg/d.

In summary, nortriptyline did not improve pain and quality of life in patients with peripheral neuropathic symptoms caused by platinum chemotherapy, but it improved patients' sleep. The changes in daily life scores impacted by pain in the nortriptyline and placebo groups were −0.3 and 0.2, respectively, in phase I of this crossover study (*p* = 0.04), and the change in sleep time in the nortriptyline and placebo groups were 0.5 and −0.3 (*p* = 0.02), respectively, in phase I of this crossover study. This improvement was most likely due to its adverse effect on sleepiness. The incidence of somnolence was 64% and 41% in the nortriptyline arm and placebo arm, respectively.

Frequently occurred adverse events were sleepiness (64% vs. 41%, *p* = 0.09), dry mouth (83% vs. 46%, *p* = 0.001), dizziness (49% vs. 15%, *p* = 0.002), impaired thinking (23% vs. 12%, *p* = 0.34), and constipation (54% vs. 34%, *p* = 0.1) in the nortriptyline arm and placebo arm, respectively.

#### 3.4.4 Amitriptyline

Amitriptyline was explored in an eight-week double-blinded randomized controlled trial conducted in 2008 in Finland (Kautio et al., 2008). The sample size was 33, and patients developed CIPN due to taxanes, platinum, or vinblastine chemotherapy. Amitriptyline capsules started at 10 mg/d and gradually increased to 50 mg/d. Compared with the placebo arm, only the Quality of Life measured by the global health score, EORTC QLQ-C30 was improved (*p* = 0.038) in the amitriptyline arm. Although amitriptyline reduced the number of times patients woke up during the night (9 vs. 5, amitriptyline vs. placebo, respectively), it was not statistically significant. The duration of sleep did not change significantly in either group. Furthermore, there were no statistically significant differences in the severity of the neuropathic symptoms, physical activity, depression scale and global improvement between the two groups. Similar to nortriptyline, drowsiness was one of the main adverse reactions with amitriptyline. Nortriptyline is an active metabolite of amitriptyline.

#### 3.4.5 KA cream

A study investigated the effect and adverse events of KA (ketamine and amitriptyline) topical application for CIPN in 462 participants (Gewandter et al., 2014). KA cream was topically applied at the maximum dose of 4 g twice daily. The overall course of interventions was 6 weeks. A statistically insignificant effect of KA cream in relieving CIPN-related pain (the change of mean pain score, −0.208, 95%Cl, −0.694 to 0.278, *p* = 0.4) was observed. However, patients in the taxane arm experienced greater pain relief than those in the non-taxane arm (the change mean pain score: 0.398, 95%Cl, -0.782 to -0.015, *p* = 0.042). The topical application of KA cream was also well tolerated.

#### 3.4.6 BAK pluronic lecithin organogel

One study investigated the effect and tolerance of BAK pluronic lecithin organogel (BAK-PLO; 1.31 g compound gel containing 10 mg baclofen, 40 mg amitriptyline hydrochloride, and 20 mg ketamine) for CIPN symptoms in 203 participants (Barton et al., 2011). BAK-PLO was applied topically twice daily. The duration of treatment was 4 weeks. Sensory neuropathic symptoms tended to improve. The mean changes in the sensory neuropathy subscale from baseline to 4 weeks were 8.1 ± 15.05 in the BAK arm and 3.8 ± 15.52 in the placebo arm (*p* = 0.053). However, BAK-PLO did not improve the CIPN-related pain, reduce the incidence of adverse events or improve the Profile of Mood States (POMS) score. The POMS evaluated the current or recent emotional state, such as tension, depression, anger, energy, fatigue, and confusion.

#### 3.4.7 Topical citrullus colocynthis (bitter apple) application

The effect and safety of topical *Citrullus colocynthis* (bitter apple) were evaluated in a double-blinded RCT for CIPN symptoms in 32 participants (Rostami et al., 2019). The topical preparation was applied locally on hands and feet twice daily, 2 ml each time. The study period was 4 weeks, and the Functional Assessment of Cancer Therapy/Gynecologic Oncology Group-Neurotoxicity (FACT/GOG-Ntx) score was the only curative indicator analyzed. At the end of treatment, no significant improvement was observed in this index and the incidence of adverse events. The total score of the FACT/GOG-Ntx scale was 1.05 ± 1.36 and 2.40 ± 1.90 (*p* = 0.879) in the intervention and placebo groups, respectively.

#### 3.4.8 Crocin

Crocin was investigated in an 18-week open-labeled, randomized, controlled, crossover study specific to the curative effect of CIPN through the scores of pain, quality of life, ENS, and sensory neuropathy (Bozorgi et al., 2021). The total sample size was 177. Crocin tablets that were derived from traditional Persian medicine were given at a dose of 15 mg twice daily (each crocin tablet contains 15 mg of crocin). Compared with the placebo arm, crocin relieved the symptoms of CIPN, such as pain, paresthesia, and depression. Furthermore, crocin improved the quality of life. Compared with the placebo arm, the changes in mean scores in the crocin arm were -2.5 for NRS mean pain (*p* = 0.002), -0.4 for BPI (*p* = 0.009), -8.3 for McGill pain rating index (*p* = 0.005), −0.04 for ENS (*p* = 0.007), −0.8 for NCIC-CTC scale (*p* = 0.005), -0.8 for WHO scale (*p* = 0.003), −7.2 for SDS (*p* = 0.009), −0.9 for NPS (*p* = 0.005), +0.6 for SGIC (*p* < 0.005), and +8.1 QOL scales (*p* = 0.009). However, the number of withdrawals and the incidence of adverse events in patients treated with crocin were slightly higher than those treated with a placebo (15.7% vs. 8%, *p* = 0.12). The most common adverse events were grade 1 (except nausea) and were increased appetite (14.7%; 2.9%), sedation (8.8%; 5.8%), headache (8.8%; 5.8%), nausea (8.8%; 2.9%), hypomania (5.8%; 5.8%), stomachache (5.8%; 2.9%), vomiting (2.9%; 2.9%), and swelling of feet (2.9%; 0%) in the crocin and placebo groups, respectively.

#### 3.4.9 Tetrodotoxin

Tetrodotoxin (TTX) was investigated in a double-blinded randomized controlled trial with a sample size of 51, conducted in 2021 in the United States (Goldlust et al., 2021). CIPN symptoms were caused by taxanes and platinum compounds. Tetrodotoxin was given 30 µg subcutaneously twice daily and the duration of treatment was 4 weeks. TTX improved the pain symptoms in patients with CIPN. Compared with the placebo, TTX made a statistically significant improvement in the SF-36 body pain score (*p* = 0.004), the EORTC CIPN20 sensory symptom subscale (*p* = 0.091), and physical component subscales (*p* = 0.076) on day 28. The change in mean pain was a -1.5 ± 1.8 score in the fourth week, and pain relief was best by 3 weeks. Meanwhile, TTX at 30 µg bid achieved 30% pain relief in 30.8% of patients during the first week of treatment and 38.5% of patients on day 28. However, most patients (80%–92.3%) experienced more than one AE due to TTX. The incidence of adverse reactions, such as oral paresthesia, oral hypoesthesia, headache, dizziness, nausea, and limb pain was higher than placebo (oral paresthesia, 42.3% vs. 16.0%; oral hypoesthesia, 38.5% vs. 20%; paresthesia, 26.9% vs. 20%; headache, 34.6% vs. 24%; dizziness, 30.8% vs. 12%; fatigue, 11.5% vs. 16%; nausea, 23.1% vs. 4%; limb pain, 11.5% vs. 4%).

#### 3.4.10 Acetyl l-carnitine

A study investigated the effect and tolerance of acetyl l-carnitine (ALC) in 462 participants who developed CIPN symptoms caused by taxanes, platinum, or vinblastine (Sun et al., 2016). This was an 8-week study in which ALC enteric-coated tablets were given 1 g twice daily. At the end of treatment (8 weeks after the onset of intervention), the neurotoxicity was improved in 50.5% of the patients in the ALC arm, compared with a 24.1% reduction in the placebo arm (95%Cl, 14.1%–38.5%, *p* < 0.001). Only the Nerve conductive velocity (NCV) of the sural nerve was significantly different between the ALC and placebo groups. Other neurological NCV tests found no difference between the two groups. Additionally, ALC therapy significantly improved the NCV in the ALC arm (60.7%), compared with the placebo arm (56.9%; *p* < 0.05). ALC treatment also reduced cancer-associated fatigue, and the difference was significant between the two arms on week 8 (33.7% vs. 18.5%, *p* = 0.014) and week 12 (41.1% vs. 25%, *p* < 0.015) through PPS (per protocol set). However, the difference was not statistically significant after 8 weeks of treatment between the ALC (31.2%) and placebo (19.8%) groups (*p* = 0.0501) through the full analysis set (FAS). Compared with the placebo group (13.0%), ALC caused a statistically significant improvement in Karnofsky physical score (KPS) (29.3%; *p* < 0.05). ALC had no severe adverse reactions. The common adverse events were gastrointestinal reactions such as vomiting, abdominal distension, and diarrhea. Also, no significant difference in the incidence of adverse events was found between the ALC (19.5%) and placebo (15.3%) groups (*p* > 0.05).

#### 3.4.11 Monosialotetrahexosyl ganglioside

Zhou et al.‘s study (Zhou et al., 2021) was the first study of the use of GM1 for the treatment rather than prevention of Oxaliplatin-induced peripheral neuropathy (OIPN). GM1 improved the symptoms of OIPN, such as pain and neurotoxicity, and GM1 was well tolerated. MCIPN, a new author-defined patient reported outcome measure indicator (≥30% improvement for the relief of neurotoxicity) modified EORTC QLQ-CIPN20, was used for neurotoxicity in patients with CIPN. A 30% improvement was considered a response and a 30%–50% improvement was considered a high response (MCIPN responders: 53% vs. 14%, *p* < 0.0001; VAS responders: 49% vs. 22%, *p* = 0.001; double responders: 41% vs. 7%, *p* < 0.0001; high responders: 32% vs. 13%, *p* = 0.004).

#### 3.4.12 Duloxetine

The efficacy of duloxetine in relieving CIPN symptoms was studied in 5 RCTs (Smith et al., 2013; Hirayama et al., 2015; Avan et al., 2018; Farshchian et al., 2018; Salehifar et al., 2020). In all five elucidations, duloxetine was effective in the treatment of CIPN. Among these studies, four studies were head-to-head trials in which duloxetine was compared to pregabalin (Avan et al., 2018; Salehifar et al., 2020), VB12 (Hirayama et al., 2015), and venlafaxine (Farshchian et al., 2018). The remaining literature (Smith et al., 2013) was the first to report the efficacy of duloxetine in the treatment of CIPN, and duloxetine relieved pain from CIPN, compared with the placebo arm. Furthermore, duloxetine was more effective for CIPN caused by platinum compounds than taxane compounds. Hirayama et al. (2015) conducted an open-labeled, randomized, crossover study with duloxetine for CIPN, and duloxetine reduced pain caused by CIPN compared with VB12 in Japanese patients. In a study published in 2018, similar to duloxetine, pregabalin improved pain, overall health, and quality of life of patients. Interestingly, pregabalin was more effective than duloxetine in improving pain (*p* < 0.001) and insomnia (*p* < 0.001). However, improvement in the emotional functioning score was found only in the duloxetine arm (*p* < 0.001) (Avan et al., 2018). A RCT published in 2020 similarly concluded that pregabalin was more effective than duloxetine in the treatment of CIPN (Salehifar et al., 2020). Both venlafaxine and duloxetine reduced pain and symptoms of sensory and motor neuropathies in patients with CIPN, and venlafaxine improved hypertension. The hypertension frequency was 51.9% vs. 86.5% vs. 81.4% in venlafaxine, duloxetine, and placebo arms at week 4, respectively (*p* < 0.001). Furthermore, venlafaxine reduced hypertension frequency (*p* < 0.05) (Farshchian et al., 2018). Therefore, venlafaxine might be beneficial in patients with CIPN and hypertension. But generally, duloxetine was more effective than venlafaxine. The detailed results of the above studies are found in Supplementary Table S1.

#### 3.4.13 Venlafaxine

Venlafaxine was investigated in a three-arm, double-blinded, randomized controlled trial of venlafaxine, duloxetine, and placebo with a total sample size of 156 and CIPN caused by taxane or platinum (Farshchian et al., 2018). The study lasted 4 weeks and was published from Iran in 2018. The dose of duloxetine was 30 mg/d, and the dosage of venlafaxine was 37.5 mg/d. Both venlafaxine and duloxetine improved the symptoms of CIPN. But only venlafaxine improved hypertension (Refer to Section 3.4.12 and Supplementary Table S1 for details).

#### 3.4.14 Pregabalin

Pregabalin was investigated in 3 RCTs, and 2 of them compared pregabalin to duloxetine. The dosage and duration of treatment were consistent in both studies (pregabalin 75 mg/d at week 1 and 75 mg bid during week 2–6; duloxetine 30 mg/d at week 1 and 30 mg bid during week 2–6), and the sample size was 82 in both groups (Avan et al., 2018; Salehifar et al., 2020). However, pregabalin was compared with gabapentin in another 8-week study with a sample size of 63, and patients were given 75 mg of pregabalin orally twice daily or 300 mg of gabapentin orally twice daily (Manjushree et al., 2018). Manjushree et al. found that (Manjushree et al., 2018) both pregabalin and gabapentin significantly alleviated the patients' pain caused by CIPN. VAS with gabapentin decreased from 8.3 ± 1.43 to 1.8 ± 2.51 at the end of treatment (*p* < 0.0001) and VAS with pregabalin decreased from 8.2 ± 1.62 to 0.8 ± 0.96 at the end of treatment (*p* < 0.0001). Generally, pregabalin was superior to gabapentin in the treatment of CIPN. However, this conclusion was inconsistent with the conclusion from the study by Rao et al. (2017). Information on the two studies is detailed in Supplementary Table S1. Similarly, two studies on pregabalin vs. duloxetine (Avan et al., 2018; Salehifar et al., 2020) were summarized in section 3.3.12. In summary, pregabalin was more effective than duloxetine and gabapentin.

## 4 Discussion

CIPN not only seriously affects the quality of life in patients but also brings additional economic burden to patients. A specific set of goals, transparent and reproducible methods, systematic and comprehensive searches, assessment of the validity of results including the risk of bias, and a systematic presentation of those results are required to evaluate the existing treatments or interventions of CIPN. ASCO presently recommends duloxetine as the sole treatment for painful CIPN. In this systematic review involving 17 RCTs, venlafaxine, pregabalin, crocin, tetrodotoxin, acetyl l-carnitine, and monosialotetrahexosyl ganglioside demonstrated some benefits in treating CIPN. However, only BAK topical analgesic gel improved CIPN symptoms without statistical significance. Before the elucidation on KA topical cream, only BAK topical analgesic gel (0.76% baclofen, 3% amitriptyline hydrochloride, and 1.5% ketamine) exhibited slight benefit, but not enough to conclude (Bozorgi et al., 2021). This might have been attributed to its lower dose and transdermal absorption. Compared with KA cream, BAK topical analgesic gel had an additional component baclofen, which might have contributed to more effectiveness of BAK compared to KA. Further, no subsequent studies on BAK were conducted. And two studies on the efficacy of gabapentin were controversial. Besides, the quality of evidence in most studies was not high, but the smaller sample size was the main problem. Therefore, we are unable to advise based on this evidence, and more future studies are needed to make definitive conclusions. Nevertheless, this systematic review can still provide reference value for conducting subsequent studies.

Based on the evidence to date, duloxetine is still effective and well-tolerated in the treatment of CIPN. However, no established standard for duloxetine in the treatment of CIPN is not presently available. From the analysis of all five RCTs included, duloxetine was beneficial for the treatment of CIPN. The dose of duloxetine ranged from 20 g/d to 60 g/d. Patients received orally 60 mg of duloxetine daily in the first published study in 2013 (Smith et al., 2013). The dosages of duloxetine in the subsequently published studies were 20 mg daily for the first week and 40 mg daily for the remainder of the study (Hirayama et al., 2015), 30 mg daily for the first week and 30 mg twice daily until 6 weeks (Avan et al., 2018), or 30 mg/d orally for 4 weeks (Farshchian et al., 2018). Although duloxetine was well tolerated without severe adverse reactions in the five RCTs analyzed a recent clinical, open-labeled experience identified the poor tolerance of duloxetine, with 20% of subjects dropping out due to lack of efficacy and 37% dropping out due to adverse events (Velasco et al., 2021). The incidence of adverse reactions (47%) and discontinuation rate (54.8%) of duloxetine were also quite high with long-term use. We included studies with treatment durations of 4–6 weeks and no studies with long-term follow-up. Further, nausea was the most commonly reported adverse effect leading to treatment discontinuation. Other common adverse reactions of duloxetine were dry mouth, insomnia, drowsiness, constipation, dizziness, and fatigue. Incidence of hepatic events such as liver injury (Kang et al., 2011; Xue et al., 2011; Malik et al., 2021), hyponatremia (Hu and Wurster, 2018; Wang et al., 2018; Ikeguchi et al., 2020), hyperprolactinemia and galactorrhea (Derle and Can, 2021), rapid eye movement sleep behavior disorder (Tan et al., 2017), weight loss (Poppen et al., 2021), and tachycardia (Stevens, 2008) were also reported due to duloxetine. Although duloxetine was well tolerated for 4–6 weeks of treatment based on the five RCTs analyzed, these adverse effects should be consistently monitored. Importantly, duloxetine needs to be discontinued slowly due to its untoward withdrawal symptoms (Fava et al., 2018). The pharmacokinetics of duloxetine also differ between specific populations. The bioavailability of duloxetine in female non-smokers is greater than that in male smokers, likely due to lower CYP1A2 enzyme activity in females than in males (Duloxetine is mainly metabolized through CYP1A2) (Knadler et al., 2011). The rate of elimination of duloxetine in elderly women (older than 65 years) is slower than that in younger women. Patients with liver insufficiency such as chronic liver disease or cirrhosis should avoid taking duloxetine due to its weak elimination ability (Knadler et al., 2011). Most of the metabolites of duloxetine (70%) were excreted in the urine. On population pharmacokinetic analyses, duloxetine should be avoided in patients with end-stage renal disease and severe renal impairment (CLCR of <30 ml/min), but it does not need to be adjusted in patients with mild-to-moderate renal impairment (CLCR of 30 and 80 ml/min) (Knadler et al., 2011). Duloxetine with FDA Grade C for pregnancy appeared to be safe for pregnant women. Two observational studies conducted in Sweden and Denmark demonstrated no increased risk of congenital malformations or stillbirth (Ankarfeldt et al., 2021a) and spontaneous or elective abortion (Ankarfeldt et al., 2021b), respectively. However, when the advantages outweigh the disadvantages, it can be used in pregnancy. But no pregnant women received chemotherapy because of the high teratogenicity, carcinogenicity, and mutagenicity of chemotherapeutic drugs. Duloxetine is mainly metabolized by CYP1A2 and CYP2D6, and duloxetine enteric-coated tablets can be affected by gastrointestinal PH. Besides, caution should also be taken for the possible occurrence of drug interactions when duloxetine is accompanied by alcohol or high plasma protein-binding drugs. The drug interactions between duloxetine and some specific drug can be referred to in this literature (Knadler et al., 2011).

Pregabalin was more effective than duloxetine in treating CIPN (Salehifar et al., 2020) and improved insomnia in patients, and duloxetine improved patients’ mood (Avan et al., 2018). But only two randomized placebo-controlled trials investigating pregabalin for the prevention of CIPN were identified and no benefits were observed for the prevention of CIPN (Shinde et al., 2016; de Andrade et al., 2017). But two RCTs investigating gabapentin had the opposite conclusion. Furthermore, although ALC was beneficial with an 8-week treatment (Sun et al., 201624 weeks of ALC therapy significantly worsened the CIPN symptoms in a long-term follow-up analysis over 2 years (Hershman et al., 2018). Therefore, future studies should be considered to draw firm conclusions.

Besides, standardized diagnostic criteria, study design, outcome indicators, and outcome measurement methods were lacking in these published studies. Due to variations in chemotherapy drugs used and outcome indicators in every study, it was difficult for us to conduct a quantitative meta-analysis. Thus, long-term studies with larger sample sizes should be implemented following a standardized study design, including the inclusion of patients, setting of outcome indicators, and validating measurement methods of outcome indicators to ensure a high degree of consistency. However, we put forward some advice about the therapeutics for chemotherapy-induced peripheral neuropathy.

First, we should focus on the basic information of patients with peripheral neuropathy, such as the chemotherapy drugs used before developing CIPN, gender, and duration of the CIPN symptoms. Since different types of chemotherapy drugs have different mechanisms of antitumor action, the mechanisms of the development of peripheral neuropathy caused by chemotherapeutics are also different. Hence, clarification of different types of chemotherapy drugs used is important to select drugs for clinical trials to alleviate CIPN. The mechanisms of the development of CIPN were quite complex and herein, a few studies were included for reference (Zajaczkowska et al., 2019; Bae et al., 2021; Burgess et al., 2021; Kang et al., 2021). In one study (Gewandter et al., 2014), although KA cream (2% ketamine and 4% amitriptyline) did not have benefits in patients with CIPN, patients in the taxane arm experienced a larger pain improvement than those in the non-taxane arm by the application of KA cream. At the same time, duloxetine showed a better analgesic effect for peripheral neuropathy induced by platinum compounds than taxane compounds (Smith et al., 2013). The chemotherapeutics that caused CIPN should be focused on the selection of drugs to alleviate CIPN. A recent observational study with a sample size of 100 found that female gender and short-lasting CIPN (<6 months) were independently associated with a favorable response to duloxetine (Velasco et al., 2021). A secondary analysis of a randomized controlled trial found that patients with better emotional states were more likely to report reduced pain from duloxetine (*p* = 0.026) (Smith et al., 2017).

Second, a set of rigorous diagnostic and evaluation criteria should be established. Currently, there are no unified diagnostic and evaluation criteria for CIPN. Similar problems existed in the studies we included in the analysis, and most studies had their own outcome metrics and measuring methods that prevented conducting a quantitative meta-analysis. Additionally, due to a common problem of small sample sizes, studies with larger sample sizes are required. Furthermore, long-term follow-up studies are also vital. Although a 12-week randomized controlled trial conducted in China found that oral administration of ALC (1000 mg three times daily) was effective in improving the symptoms of CIPN and physical conditions, and reducing cancer-associated fatigue (Sun et al., 2016), another randomized, double-blinded, multicenter study (ALC 1000 mg three times daily) in women undergoing adjuvant taxane-based chemotherapy for breast cancer found that 24 weeks of ALC therapy resulted in statistically significant worsening of CIPN over 2 years (Hershman et al., 2018).

Finally, in addition to drug therapy, some non-drug areas, such as physical therapy and traditional natural medicines should be focused to use as potential candidates for the treatment of CIPN. The effectiveness of crocin for the treatment of CIPN also provided us a hint to find some other traditional natural medicines to treat CIPN. A recent systematic review and meta-analysis of Chinese herbal medicine found that topical application of Chinese herbal medicine was effective in treating CIPN as it significantly improved clinical symptoms and quality of life in patients with CIPN (Li et al., 2022). Crocin was derived from Saffron, a traditional Persian medicine (TPM), which had analgesic, antioxidant, anti-genotoxic, anti-tumor, anti-inflammatory, anticonvulsant, anti-depressant, antibacterial, sedative, memory-enhancing, and neuroprotective effects (Bozorgi et al., 2021).

## 5 Conclusion

The primary objectives for this systematic review were to examine the efficacy of drugs in the treatment of CIPN using existing randomized controlled trials to provide evidence for clinical practice and future studies. The analysis results demonstrated that pregabalin, crocin, tetrodotoxin, venlafaxine, and GM1 may be beneficial for the treatment of CIPN in addition to duloxetine. ALC and gabapentin are somewhat controversial in treating CIPN. However, the number of randomized controlled trials of CIPN treatment is small and most studies are lacking evidence to provide a solid basis for decision-making. Therefore, a standardized study design involving the characteristics of patients, the duration of therapy, and outcome indicators is required. RCTs with larger sample sizes and longer follow-ups are recommended to comprehensively evaluate the efficacy of the drugs in the treatment of CIPN. Finally, some randomized controlled trials investigating the curative effect of peri-neural platelet-rich plasma injection, donepezil, topical menthol application, topical cannabidiol, and single-cycle tetrodotoxin for the treatment of CIPN are expected to be carried out.

## Data Availability

The original contributions presented in the study are included in the article/Supplementary Material, further inquiries can be directed to the corresponding author.
