# Supplementary material for: Treatment for chemotherapy-induced peripheral neuropathy: A systematic review of randomized control trials
Source: Front Pharmacol. 2022 Dec 23;13:1080888. doi: 10.3389/fphar.2022.1080888 (PMC9822574; doi:10.3389/fphar.2022.1080888)
Supplement: Supplementary file 1 [file Table1.pdf]

TABLE S1 Description of study intervention and outcomes

Included Studies	Intervention		Intervention time (Weeks)	Outcome	Results
	Treatment	Control			
Rao et al. 2007 crossover, open	Gabapentin capsules were started at 300mg/d and increased to 2700mg/d within 3 weeks	Placebo	14	1. Numerical Rating Scale (NRS), average pain<br>2. Brief Pain Inventory Short Form (BPI-SF), average pain<br>3. The Short Form-McGill Pain Questionnaire to assess different characteristics of neuropathic pain such as throbbing, gnawing, shooting, aching, and burning<br>4. The 0-3 the Eastern Coopera-tive Oncology Group (ECOG) neuropathy scale (ENS) average pain<br>5.Quality-of-life (QOL) Uniscale (a single-item measurement of global QOL on a	No observed benefits from treatment.

				numeric analog scale from 0 to 100)<br>6. Incidence of adverse events<br>7. World Health Organization neuropathy score<br>8. Total Symptom Distress Scale (SDS) score, mean<br>9. Opioids use<br>10. Nonopioid analgesics used<br>11. Subject global impression of change	
Rao et al. 2008 double-blind	Lamotrigine, 25 mg for 2 weeks, then 25 mg, Bid, for 2 weeks, then 50 mg, Bid, for 2 weeks, then	Placebo	10	1. NRS average pain<br>2. BPI average pain<br>3. McGill pain rating index<br>4. Eastern Cooperative Oncology Group Neuropathy average pain Scale<br>5. QOL Uniscale<br>6. Incidence of adverse events<br>7. SDS score, mean	No overall benefit from treatment.

	100 mg, Bid, for 2 weeks, and then 150 mg, Bid, for 2 weeks.			8. Subject global impression of change	
Lavoie Smith et al. 2013 crossover, open	Duloxetine 60mg, qd, P.O.	Placebo	12	1. BPI<br>2. Pain interference with daily function<br>3. Quality of life<br>4. Incidence of adverse events	Duloxetine was benefit for CIPN treatment.<br>At the end of the first phase of the crossover study,<br>1.The decrease in average pain score<br>Duloxetine (1.06; 95% CI, 0.72-1.40) was superior to placebo (0.34; 95% CI, 0.01-0.66; P=0.003), namely the mean change of the mean pain score between two arms was 0.73 (95% CI, 0.26-1.20).<br>2. Compared with placebo, RR value<br>30% pain decrease: duloxetine, 1.96 (95% CI, 1.15-3.35); 50% pain decrease: 2.43 (95% CI, 1.11-5.30;<br>30% pain reduction in patients treated with platinum: 3.05 (95% CI, 1.49-6.27); 50% reduction: 3.78 (95% CI, 1.32-10.84), however, 30% pain decrease in patients treat with taxane: 0.97 (95% CI, 0.41-2.32);

					50%: 1.22 (95% CI, 0.35-4.18).<br>3. The change of mean interference score<br>duloxetine, 7.9 (95% CI, 5.4-10.5); placebo, 3.5 (95% CI, 1.1-5.9). The mean change between the 2 arms was 4.40 (95% CI, 0.93-7.88).<br>4. The mean difference in the FACT/GOG-Ntx total score<br>Duloxetine: 2.44 (95% CI, 0.43-<br>4.45); placebo: 0.87 (95% CI, 1.09-2.82). The mean change between the 2 arms in mean change score was 1.58 (95% CI, 0.15-3.00, P=0.03).<br>Although the incidence of adverse events was similar in both arms, the rate of withdrawal due to adverse events was significantly higher in the duloxetine arm than in the placebo arm.
Gewandter et al. 2014 double-blind	KA cream, apply topically, maximum 4g each time, Bid	Placebo	6	1. NRS<br>2. Incidence of adverse events	No observed benefits from KA cream treatment. And no significant adverse effects were observed.

Hirayama et al. 2015 crossover, open	Duloxetine, P.O.<br>First week: 20 mg/day, Then 40 mg/day	VB_{12} 1.5mg, qd, P.O.	10-14	1. Visual Analogue Scale/Score (VAS) pain score<br>2. Incidence of adverse events	There was benefit for CIPN treatment.<br>HR (the end of the first phase of the crossover study): 1. Nonattainment of a 30 % reduction in numbness and pain were 0.25 in duloxetine arm, 0.4 in VB_{12} arm. 2. Nonattainment of a 50 % reduction were 0.28 in duloxetine arm and 0.25 in VB_{12} arm.<br>No adverse reactions of more than two grades were found. Insomnia was the common adverse even in duloxetine group. 5 people dropped out of the study because of the adverse events of duloxetine (3 fatigue, 1 nausea, 1 somnolence).
Manjushree et al. 2021 open	Gabapentin, 300 mg, Bid, P.O.	Pregabalin 75 mg, Bid, P.O.	8	1. VAS<br>2. Rescue medications were required<br>3. Incidence of adverse events	Pregabalin and gabapentin both all can improve CIPN. And pregabalin might be more effective than gabapentin.
Bozorgi et al. 2021 crossover, open	Crocin tablets, 15 mg, bid, P.O. (each crocin	Placebo	18	1. NRS<br>2. BPI<br>3. McGill pain rating index<br>4. Eastern Cooperative Oncology Group	There was benefit for CIPN patients by crocin treatment.

	tablet contains 15 mg of crocin)			Neuropathy average pain Scale<br>5.NCIC-CTC (National Cancer Institute of Canada Common Toxicity Criteria)<br>6. World Health Organization scale<br>7. SDS<br>8. Neuropathy Pain Scale<br>9. SGIC (Subjective Global Impression of Change)<br>10. QOL scales<br>11. Incidence of adverse events	
Goldlust et al. 2021 double-blind	Tetrodotoxin, 30 μg, Bid, I.H.	Placebo	4	1. Numeric Pain Rating Scale<br>2. SF-36 (Short Form Health) Body Pain<br>3. European Organization for Research and Treatment of Cancer Chemotherapy-Induced Peripheral Neuropathy 20 sensory symptom subscale<br>4. the Physical Component subscales	There was benefit in improving pain in patients with CIPN.

				5. Incidence of adverse events	
Hammack et al. 2002 crossover, double-blind	Nortriptyline tablets started at 25mg/d and could be increased by 25mg/d every other week for a maximum dose of 100mg/d	Placebo	9	1. VAS pain scale (scaled from 0 to 100)<br>2. QOL (scaled from 0 to 100)<br>3. Satisfaction with treatment<br>4. Pain affects daily life<br>5. Hours of sleep<br>6. Incidence of adverse events	No observed benefits from treatment.
Kautio et al. 2008 double-blind	Amitriptyline capsules started at 10mg/d and gradually	Placebo	8	1. Quality of Life<br>2. The number of nightly awakenings<br>3. the severity of the neuropathic symptoms<br>4. Physical activity<br>5. Depression scale	No observed benefits from treatment.

	increased to 50mg/d			6. Global improvement	
Barton et al. 2011 double-blind	BAK-PLO, Apply topically, Bid	Placebo	4	1. The mean changes of the sensory neuropathy, motor neuropathy subscale and the autonomic subscale.<br>2. BPI<br>3. Profile of Mood States<br>4. Incidence of adverse events	No observed benefits from treatment.
Sun et al. 2016 double-blind	Acetyl L-Carnitine enteric coatel tablets, P.O. 1g/time, Bid	Placebo	8	1. Neurotoxicity<br>2. Electrophysiological examination<br>3. Evaluation of electrophysiology<br>4. Cancer-associated fatigue<br>5. Karnofsky physical score<br>6. Incidence of adverse events	There was benefit for CIPN treatment (Sun et al., 2016) but subsequent studies have been controversial (Hershman et al., 2018).
Avan et al. 2018 double-	Pregabalin 75mg/d, at week 1 and	Duroxetine 30mg/d, at week 1 and	6	1. QLQ scores<br>2. Pain score<br>3. Insomnia score	Benefit was observed from pregabalin and duloxetine for CIPN treatment. And pregabalin was more effective in decreasing of pain score than duloxetine.

blind	75mg, Bid, during week 2-6	30mg, Bid, during week 2-6		4. Emotional functioning score	The global health status/QOL scale significant improved after 6 weeks (P=0.002) in each treatment group. However, there was no significant difference (P=0.91) between pregabalin arm and duloxetine arm.<br>After six weeks, both pregabalin and duloxetine improved pain symptoms (mean pain score, P＜0.001), and pregabalin had better analgesia than duloxetine ((P<0.001)). Pregabalin improved insomnia measured by insomnia score (P＜0.001), while duloxetine improved mood measured by emotional functioning score (P＜0.001).<br>Somnolence (22.5% VS 4.8%, P=0.02) and dizziness (17.5% VS 0%, P=0.005) were more common in pregabalin arm, whereas nausea/vomiting (9.5% VS 0%, P=0.04) was more common in duloxetine arm.
Farshchian et al. 2018 double-blind	Duloxetine 30mg/d Venlafaxine 37.5mg/d	Placebo	4	1. Hypertension frequency<br>2. Cranial neuropathy grade<br>3. Sensory neuropathy, motor neuropathy<br>4. Neuropathic pain<br>5. Incidence of adverse events	In brief, both duloxetine and venlafaxine all can improve symptoms in patients with CIPN. And the two drugs were well tolerated.<br>At week 4, compared with placebo, both duloxetine and venlafaxine all improved the patient's symptoms of cranial neuropathy, sensory and motor neuropathy, and pain (P＜0.05). Only venlafaxine reduced the

					incidence of high blood pressure (P＜0.05).<br>No drug-related withdrawals were reported. The common adverse events were nausea, constipation and insomnia in venlafaxine arm and dizziness, fatigue and headache in duloxetine arm.
Rostami et al. 2019 double-blind	Topical Citrullus colocynthis oil, 2ml, apply topically on the plantar and dorsal surface of the hands and feet, Bid	Placebo	4	1. Functional Assessment of Cancer Therapy/Gynecologic Oncology Group-Neurotoxicity score	No observed benefits from treatment.
Salehifar et al. 2020	Pregabalin 75mg/d, for	Duloxetine, P.O.	6	1. VAS<br>2. National Cancer Institute Common	In brief, pregabalin was more effective than duloxetine and adverse effects (Adverse events) of two drugs were mild and well-tolerated.

double-blind	week 1 and 75mg, Bid, during week 2-6	First week: 30 mg/day; then 30 mg, Bid.		Terminology Criteria for Adverse Events. grade<br>3. PNQ, Patient Neurotoxicity Questionnaire score<br>4. Graded improvement rate of TIPN<br>5. Adverse events	VAS score decreased from baseline (65.65 ± 8.78, 62.43 ± 9.18) to week 6 (31.65 ± 8.30, 43.36 ± 5.75) in pregabalin arm and duloxetine arm, respectively (P＜0.001).<br>NCI-CTCAE grade decreased from baseline (2.08 ± 0.47, 1.90 ± 0.53) to week 6 (1 ± 0.00, 1.60 ± 0.50) in pregabalin arm and duloxetine arm, respectively (P＜0.001).<br>PNQ score decreased from baseline (3.30 ± 0.46, 3.33 ± 0.48) to week 6 (2.18 ± 0.38, 3.02 ± 0.64) in pregabalin arm and duloxetine arm, respectively (P＜0.001).<br>The response rates were 92.5% in pregabalin arm VS 38.1% in duloxetine arm, respectively (p<0.001) at week 6.<br>The improvement in sensory neuropathy measured by the NCI-CTCAE scale: 37/40 (92.5%) in the pregabalin arm VS 13/42 (31%) in duloxetine arm, respectively (p<0.001) at week 6.<br>TIPN was improved from grade 3 to grade 2: 0% in pregabalin arm VS 9.5% in duloxetine arm. TIPN symptom was improved from grade 3 to grade 1: 15% in pregabalin arm VS 0% in duloxetine arm. TIPN

					symptom was improved from grade 2 to grade 1: 77.5% in pregabalin arm VS 21.5% in duloxetine arm.<br>After 6 weeks, improvement in sensory neuropathy, based to the sensory PNQ scale: 90% in pregabalin arm VS 31% in duloxetine arm ($p<0.001$).<br>Somnolence and dizziness were more frequently reported by patients with pregabalin ($p = 0.02$ and 0.005, respectively) than patients treated with duloxetine, whereas nausea/vomiting were more frequently reported in duloxetine arm ($p=0.04$). Secondly, pregabalin group was also common for adverse events of ataxia ($P=0.14$), diplopia ($P=0.3$), duloxetine group commonly reported insomnia ($P=0.16$).
Zhou et al. 2021	GM1, 60 mg/day or 40 mg/day	Placebo	6	1. The response of MCIPN (MCIPN, a newly developed patient reported outcome measure, ⩾30% improvement for the relief of neurotoxicity)<br>2. The response of VAS<br>3. A neurotoxicity improvement of ≥ 1 grade<br>4. Adverse events	GM1 reduced the severity of chronic OIPN and was well tolerated.
